# Effects of anti‐inflammatory drugs on the expression of tryptophan‐metabolism genes by human macrophages

**DOI:** 10.1002/JLB.3A0617-261R

**Published:** 2018-01-26

**Authors:** Tim Regan, Andrew C. Gill, Sara M. Clohisey, Mark W. Barnett, Carmine M. Pariante, Neil A. Harrison, MRC Immunopsychiatry Consortium, David A. Hume, Edward T Bullmore, Tom C. Freeman

**Affiliations:** ^1^ The Roslin Institute and Royal (Dick) School of Veterinary Studies University of Edinburgh Easter Bush Edinburgh Scotland UK; ^2^ School of Chemistry Joseph Banks Laboratories University of Lincoln, Green Lane Lincoln Lincolnshire UK; ^3^ Stress Psychiatry and Immunology Department of Psychological Medicine Institute of Psychiatry Kings College London London UK; ^4^ Brighton and Sussex Medical School University of Sussex Brighton UK; ^5^ Department of Psychiatry University of Cambridge Cambridge UK; ^6^ Cambridgeshire & Peterborough NHS Foundation Trust Cambridge UK; ^7^ ImmunoPsychiatry Immuno‐Inflammation Therapeutic Area Unit GlaxoSmithKline R&D Stevenage UK

**Keywords:** anti‐inflammatory, depression, inflammatory signaling, kynurenine, macrophage, monoaminergic, transcriptomics, tryptophan

## Abstract

Several lines of evidence link macrophage activation and inflammation with (monoaminergic) nervous systems in the etiology of depression. IFN treatment is associated with depressive symptoms, whereas anti‐TNFα therapies elicit positive mood. This study describes the actions of 2 monoaminergic antidepressants (escitalopram, nortriptyline) and 3 anti‐inflammatory drugs (indomethacin, prednisolone, and anti‐TNFα antibody) on the response of human monocyte‐derived macrophages (MDMs) from 6 individuals to LPS or IFN‐α. Expression profiling revealed robust changes in the MDM transcriptome (3294 genes at *P < 0.001*) following LPS challenge, whereas a more limited subset of genes (499) responded to IFNα. Contrary to published reports, administered at nontoxic doses, neither monoaminergic antidepressant significantly modulated the transcriptional response to either inflammatory challenge. Each anti‐inflammatory drug had a distinct impact on the expression of inflammatory cytokines and on the profile of inducible gene expression—notably on the regulation of enzymes involved in metabolism of tryptophan. Inter alia, the effect of anti‐TNFα antibody confirmed a predicted autocrine stimulatory loop in human macrophages. The transcriptional changes were predictive of tryptophan availability and kynurenine synthesis, as analyzed by targeted metabolomic studies on cellular supernatants. We suggest that inflammatory processes in the brain or periphery could impact on depression by altering the availability of tryptophan for serotonin synthesis and/or by increasing production of neurotoxic kynurenine.

**Abbreviations**
COX: cyclooxygenase
MDD: major depressive disorder
MDMs: monocyte‐derived macrophages
SSRI: selective serotonin reuptake inhibitors
TCA: tricyclic antidepressant

## 1 INTRODUCTION

Autoimmune and inflammatory diseases are commonly associated with mood disorders and several lines of evidence indicate inflammation may give rise to or exacerbate them.^1^, ^2^ Studies of animals exposed to proinflammatory challenges, ranging from LPS administration to social defeat, reveal that activation of the peripheral innate immune system causes a depression‐like syndrome of illness behavior: social withdrawal, reduced mobility/energy, sleep disturbance, weight loss, and anhedonia.^3^, ^4^, ^5^ In humans, IFNα‐based therapy for hepatitis C infection can trigger a major depressive disorder (MDD) resulting in around 30% of patients withdrawing from treatment.^6^, ^7^ Conversely, TNFα blockade improves depressive symptoms in patients with rheumatoid arthritis^8^ and in a subgroup of patients with MDD and elevated levels of C‐reactive protein (an acute phase protein).^9^

One plausible sequence of events is that peripheral proinflammatory cytokines such as TNF‐α, IL‐1β, and IL‐6, induced the expression of enzymes involved in tryptophan catabolism, for example, indoleamine dioxygenase, kynurenine hydroxylase, and kynureninase,^10^ thereby reducing the availability of synaptic serotonin^11^, ^12^, ^13^; which is a proximal cause of depressive symptoms.^14^ Inflammation‐induced changes in tryptophan metabolism can also lead to increased synthesis of kynurenine and its metabolites, many of which are known to be glutamatergic agonists and/or neurotoxic.^15^ Thus, inflammation could produce the “double hit” both reducing the availability of serotonin and increasing production of kynurenine.

The same mechanisms may explain the clinical observation that patients with depressive symptoms in the context of peripheral inflammation (“inflamed depression”) are less responsive to monoaminergic antidepressant drugs, so called “treatment‐resistant depression.”^16^ Selective serotonin reuptake inhibitors (SSRIs), for example, are thought to increase the synaptic availability of serotonin (by blocking active uptake by presynaptic neurons expressing the serotonin transporter protein (SERT/SLC6A4)).^17^ If synaptic serotonin is reduced by an inflammatory response, and SSRIs are not anti‐inflammatory,^18^ SSRIs are likely to be less effective in the presence of an inflammatory stimulus. Additionally, the neurotoxic effects of kynurenine and its metabolites, which are exacerbated by inflammation, are mediated by glutamatergic‐related mechanisms that are not modulated by monoaminergic antidepressants. Treatment‐resistant depression—due to reduced serotonin availability and increased neurotoxicity by non‐serotonergic mechanisms—is thus predictable in the context of peripheral inflammation.^12^, ^13^, ^19^, ^20^

Here we report on a series of experiments designed to test the mechanistic connections between inflammatory and monoaminergic systems in a human primary cell model, monocyte‐derived macrophages (MDMs). Macrophages are both initiators and mediators of inflammation‐associated pathology^21^ and have long been implicated in inflammation induced depression,^20^ largely based on the inflammatory induced activation of tryptophan depletion pathways.^10^, ^19^, ^20^ Microglia, the macrophages of the brain,^22^ play a crucial role in neuronal homeostasis, and the impairments in neuronal function associated with many clinical disorders, including major depression.^23^ Unlike human macrophages, mouse macrophages do not induce tryptophan uptake or enzymes associated with tryptophan metabolism in response to proinflammatory signals,^24^ making them a poor model for such studies. In the current study, we have used human MDMs, differentiated by CSF1, to study the impacts of 2 antidepressant drugs and 3 anti‐inflammatory drugs on the transcriptional response to 2 proinflammatory challenges: LPS and IFNα. In addition to acting as a primary human cell model for microglia, these cells are also used to examine tryptophan depletion mechanisms during peripheral inflammation, and how drugs may influence this effect.

LPS is a well‐studied Gram‐negative bacterial cell wall endotoxin that acts through activation of the TLR4 receptor, an archetypal pattern recognition receptor that signals through two well‐defined complementary signaling pathways to induce proinflammatory cytokines and IFN target genes.^25^, ^26^ As part of the FANTOM5 consortium, we have recently generated extensive promotor‐level data on the time‐course of the response of MDM.^27^ IFNα is a type‐1 IFN and was chosen as a stimulus because of the clinical data indicating that this agent can cause a depressive syndrome in patients.^6^, ^7^ We have previously compared and contrasted the transcriptional response of mouse macrophages to type‐1 IFN and LPS treatment.^28^

Three classes of anti‐inflammatory drugs were selected to examine their effects on both inflammatory stimuli: a neutralizing antibody for the inflammatory cytokine TNFα; a nonsteroidal anti‐inflammatory drug (indomethacin); and a steroidal anti‐inflammatory drug (prednisolone). Neutralizing anti‐TNFα antibodies act by binding to TNFα in circulation, thus blocking its proinflammatory effects mediated by action at TNF receptors.^29^ TNFα acts in an autocrine manner on stimulated macrophages to induce downstream targets and amplify the initial induction of proinflammatory target genes.^27^, ^30^ Indomethacin acts by inhibiting cyclooxygenase (COX) or prostaglandin synthase enzymes (COX1/COX2; PTGS1/PTGS2).^31^ The anti‐inflammatory activity of COX inhibitors is thought to depend on preventing inducible prostaglandin production, which can also act in an autocrine manner in macrophages through inducible prostaglandin receptors.^25^ Both COX‐inhibiting and TNFα‐inhibiting drugs have been shown to have some antidepressant and anxiolytic effects in patients with psychologic symptoms in the context of medical inflammatory disorders.^32^ Synthetic glucocorticoids, such as prednisolone, are amongst the most commonly prescribed anti‐inflammatory agents, and act by inducing multiple feedback repressors of inflammation, including IκB and DUSP1.^33^ The responses to glucocorticoids also differ radically between humans and mice, due to the gain and loss of glucocorticoid response elements in enhancers,^33^ so there is a clear need to study their effects in humans systems. The glucocorticoid ligand/receptor complex may also directly repress signaling by interfering with the activation of the inflammatory transcription factor NF‐κB^34^ leading to a genome wide blockade of NF‐κB interactions with chromatin.^35^

We also studied the action of two monoaminergic antidepressant drugs on MDMs, a SSRI (escitalopram) and a tricyclic antidepressant (TCA; nortriptyline). SSRIs are the most commonly prescribed drugs for treating depression and their efficacy depends at least partly on increasing synaptic availability of serotonin (5‐HT). However, 5‐HT also plays an important role in immune signaling,^36^ and SSRIs have been shown to enhance the cytolytic function of NK cells, to enhance B cell numbers, and to inhibit 5‐HT uptake and immune signaling by dendritic cells,^37^ suggesting that immune mechanisms might also contribute to their therapeutic efficacy. The TCAs are less selectively serotonergic, and also block multiple classes of receptors for acetylcholine, histamine and noradrenaline. The use of TCAs in the treatment of the residual symptoms of inflammatory bowel disease,^38^ suggests they may have some efficacy as anti‐inflammatory agents.^39^ Supplemental Fig. S1 illustrates the known mode of action of each drug examined here.

We analyzed the gene expression profiles of MDM in response to inflammatory challenge in the presence and absence of anti‐inflammatory or monoaminergic antidepressant drug treatment. Network‐based methods were deployed to represent the expression changes induced by inflammatory challenges, and the modulation of response by each of the 5 drugs tested. We tested the specific prior hypotheses (i) that proinflammatory challenges will cause changes in expression of genes related to tryptophan metabolism; (ii) that monoaminergic antidepressants (nortriptyline, escitalopram) will attenuate inflammation‐induced changes in tryptophan‐related genes; or (iii) that anti‐inflammatory drugs (prednisolone, indomethacin, anti‐TNFα antibody) will attenuate the regulation of tryptophan‐related genes; (iv) that inflammatory activation of human macrophages results in decreased production of tryptophan and increased production of kynurenine; and (v) that anti‐inflammatory drugs can attenuate changes in tryptophan metabolism and kynurenine production.

## 2 MATERIALS AND METHODS

### 2.1 Study design overview

**Figure 1:**
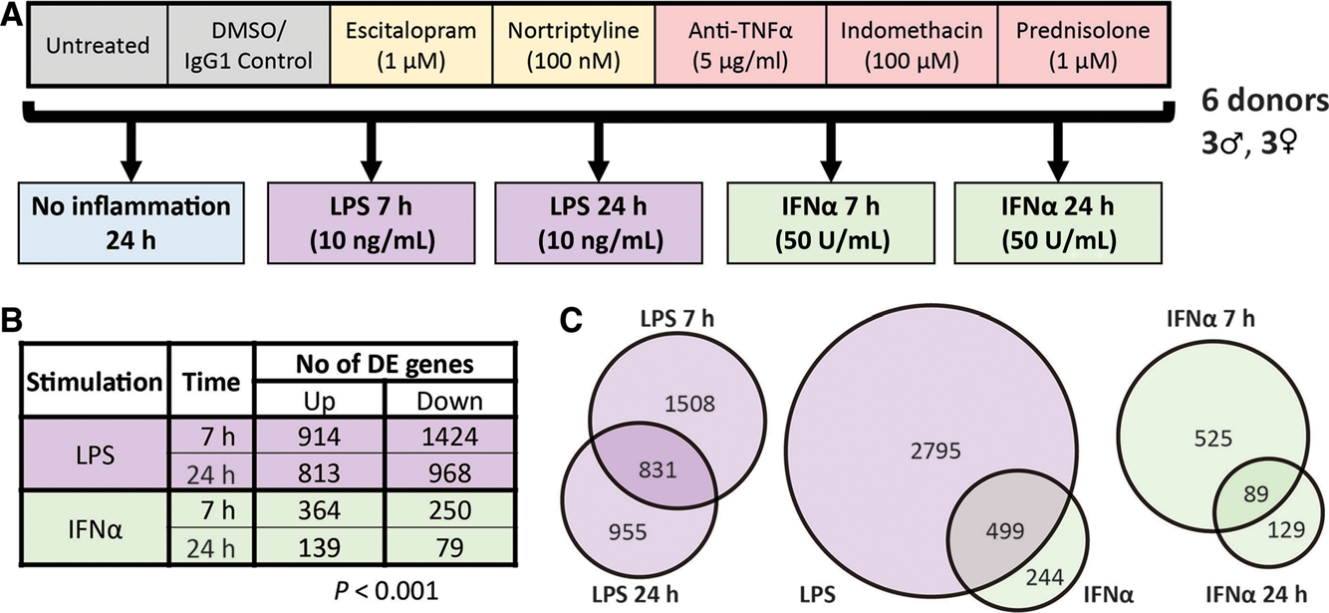
**Study design and differential gene expression in response to inflammatory challenge**. (A) Schematic representation of experimental design. Fully differentiated human MDMs (day 8) were generated from 6 individuals aged 20–30 years, 3 male and 3 female. Cells were then pretreated with either escitalopram, nortriptyline, an anti‐TNFα antibody, indomethacin, prednisolone, or controls (a nonspecific IgG antibody with DMSO vector), or untreated. Cells were then exposed to LPS or IFNα challenge and harvested at either 7 or 24 h, or cultured for 24 h with no inflammatory stimulus. (B) Numbers of significantly differentially expressed genes (DEGs) following inflammatory stimulation (*P* < 0.001). (C) Venn diagrams displaying the overlap of DEGs between IFNα and LPS challenges at early or late time points

MDM cultures from each of 6 different individuals were treated with 1 of the 3 anti‐inflammatory drugs, 2 antidepressant drugs, or a vehicle control. They were then stimulated with either IFNα or LPS for either 7 or 24 h prior to sample collection, or incubated for 24 h with no inflammatory stimulus. Cell culture supernatants were then analyzed by ELISA for cytokine production, and RNA samples extracted from cells and subjected to microarray analysis (Fig. 1). Analysis of these data revealed changes induced by inflammatory challenge in tryptophan/kynurenine gene expression that were moderated by anti‐inflammatory drugs and predictive of altered tryptophan/kynurenine metabolism. We tested this prediction in a secondary metabolomics study of the effects of inflammatory challenge and drug treatment on the levels of tryptophan and kynurenine, measured in the cell supernatant using HPLC/MS. A linear mixed effects model was employed with each donor/subject as a random effect to account for interdonor variations, which could potentially lead to false results with a sample size of only n = 6 (see below).

### 2.2 Ethics and donors

Human CD14^+^ mononuclear cells were isolated from fresh blood of volunteer donors under ethical approval from Lothian Research Ethics Committee (11/AL/0168).

### 2.3 Cell culture

Human peripheral blood monocytes were isolated from 320 mL blood samples by Ficoll gradient separation of buffy coats followed by MACS CD14^+^ selection (Miltenyi Biotec Ltd., Bisley, UK). They were then cultured at 5 × 10^5^ cells/well in 1 mL on a 12‐well plate in RPMI supplemented with penicillin/streptomycin, glutamax (Invitrogen, Loughborough, UK), and 10% fetal calf serum for 7 d in the presence of rhCSF‐1 (a gift from Chiron, Emeryville, CA) at 10^4^ U/mL to produce MDM. All donors were medically healthy and between 20 and 50 years old (3 male, 3 female).

IFNα (SRP4594; Sigma–Aldrich, Gillingham, UK) was used at 50 U/mL, within the range reported to be present in blood serum following IFNα‐2b/Ribavirin therapy for hepatitis C patients.^40^ LPS from *Salmonella enterica* serotype minnesota (Re 595, L9764; Sigma–Aldrich) was used at 10 ng/mL, which is just maximal for inducible proinflammatory gene expression.^28^ MDM cultures from each of the 6 individuals were stimulated with IFNα or LPS for 7 or 24 h, or incubated for 24 h with no inflammatory stimulus as a control. Parallel cultures included 100 μM indomethacin (Sigma–Aldrich), 1 μM prednisolone (Sigma–Aldrich), 5 μg IgG1 anti‐TNFα antibody (MAB610; R&D Systems, Wiesbaden, Germany), 100 nM escitalopram (Sigma–Aldrich), 1 μM nortriptyline (Sigma–Aldrich), or IgG1 isotype control (R&D Systems, Abingdon, UK) in DMSO at the times indicated (7 or 24 h). Data supporting the rationale and optimization of inflammatory challenge and drug doses on cell viability assays are provided in Supplemental Materials and Methods. We noted that the dose of both monoaminergic drugs (escitalopram and nortriptyline) was limited by their cytotoxic effects on MDMs in vitro (Supplemental Fig. S2).

### 2.4 Optimization of inflammatory stimulation and drug concentrations

Cell viability was measured to optimize the concentrations of drugs used (other than anti‐TNFα). This was performed using CellTiter‐Glo (Promega Ltd., Southampton, UK) according to manufacturer's instructions. Briefly, cells were seeded at 1 × 10^5^ cells/well in 96‐well, flat‐bottomed white‐walled plates (Corning Ltd., Wiesbaden, Germany) and cultured in 100 μL media. Following maturation and consequent drug treatment, 100 μL CellTiter‐Glo was added to each well (1:1 ratio) and the plate was placed on a shaker for 10 min. Luminescence was measured in technical triplicates using a GloMax^®^96‐ Microplate Luminometer (Promega) and percentage of viable cells after each treatment was calculated relative to the untreated control culture. Six biologic replicates were used to test escitalopram (SSRI) and nortriptyline (TCA), 3 for indomethacin and prednisolone. Supplemental Figs. S2A and B display the cell viability in the presence of escitalopram or nortriptyline at 24 h post‐treatment. To ensure maximal drug contact with the cells without affecting viability, concentrations of 100 nM for nortriptyline and 1 μM for escitalopram were selected for the transcriptomics studies. These concentrations are higher than peak whole blood concentrations of escitalopram (∼0.1 nM^41^) or nortriptyline (50 nM^42^) in patients. Supplemental Figs. S2C and D show cell viability was not significantly affected by any of the tested concentrations of indomethacin or prednisolone. We therefore selected concentrations of 100 μM indomethacin and 1 μM prednisolone, as used in previous in vitro studies of macrophages.^43^, ^44^ The amount of anti‐TNFα antibody used was calculated to be 5 μg/mL. This was based on the suppliers ND_50_ value of 0.01–0.04 μg/mL in the presence of 0.75 ng/mL TNFα and our own measurements of maximum TNFα production from stimulated macrophages (<30 ng/mL). We then used the following equation to calculate the amount of antibody required: $K_{D}\;=\;\;\frac{\left[\mathrm{mAb}][\;\mathrm{Target}\right]}{\left[\mathrm{Complex}\right]}$.

IFNα (SRP4594, Sigma–Aldrich) was supplemented at 50 U/mL as this concentration yields a robust inflammatory response in macrophages and is within the range reported to be present in blood serum following IFNα‐2b/Ribavirin therapy for hepatitis C patients.^40^ Although many in vitro studies typically use 100 ng/mL of LPS, we used 10 ng/mL in order to consistently stimulate the cells for 24 h without obscuring any of the subtler downstream signaling effects (LPS from *S. enterica* serotype minnesota Re 595, L9764; Sigma–Aldrich).

The final parameters for the experiment involving MDM, inflammatory stimuli and drug treatments are outlined in Supplemental Fig. 1A. Each of the MDM cultures from 6 different individuals were treated separately with each of the 3 anti‐inflammatory drugs, 2 antidepressants, vehicle control, or left untreated. Samples were then stimulated with either IFNα or LPS for either 7 or 24 h, or incubated for 24 h with no inflammatory stimulus as a control. RNA was extracted from the samples and analyzed by expression microarray

### 2.5 RNA extraction and processing

RNA was prepared using RNeasy column‐based extraction (Qiagen, Manchester, UK). 350 μL of RNeasy buffer RLT was used per sample to extract RNA, which was eluted from the column in water following on‐column DNase treatment. RNA quality was subsequently analyzed using a 2200 Tapestation (Agilent, Edinburgh, UK). For expression microarrays, 500 ng of RNA was prepared using standard Affymetrix protocols and applied to the Human Gene 2.1 ST array by Edinburgh Genomics (Edinburgh, UK).

### 2.6 Expression data analysis

Analysis was performed using R/Bioconductor packages “arrayQualityMetrics,”^45^ “oligo,”^46^ and “nlme.”^47^ Normalization was performed using RMA.^48^ Probesets were collapsed down to a single gene. These data are available on Gene Expression Omnibus (GEO GSE85333).

Differentially expressed genes (DEGs) at each of the post‐inflammatory challenge time points (LPS or IFN at 7 or 24 h) were first estimated by a linear mixed effects model with inflammatory challenge as a fixed effect and donor (participant) as a random effect. To test the hypothesis that drug treatment significantly modulated the genomic response to inflammatory challenge, we extended the linear mixed effects model to include both inflammatory challenge and drug treatment as fixed effects, the interaction between challenge and drug as a fixed effect, and donor as a random effect. This model was fit to data from the second (24 h) post‐inflammatory time point. Tests for significance of all linear model coefficients are reported at uncorrected false‐positive rates of *P* < 0.05, *P* < 0.01, and *P *< 0.001 as indicated.

Network analysis was performed using Graphia Professional software (Kajeka Ltd., Edinburgh, UK) to explore inflammation‐ and drug‐related transcriptional changes in the context of the transcriptome. Reactome software was used^49^ for enrichment analysis of DEGs previously implicated in neuronal signaling or depression.

### 2.7 Quantitation of selected neurotransmitter metabolites

To confirm the predicted metabolic effects of these treatments on the expression of genes related to tryptophan metabolism, tryptophan and kynurenine concentrations were measured by HPLC/MS in the supernatant of cells after 24 h incubation (Supplemental Materials).

### 2.8 Quantitation of cytokine production in sample supernatants

To compare protein production to transcript levels, cytokine production was measured in the supernatant of cells by use of TNF‐α (#KAC1751) and IL‐6 (#KHC0061C) ELISA kits (ThermoFisher, Runcorn, UK). Precoated plates were used according to manufacturer's instructions. Briefly, plates were blocked for nonspecific binding. Supernatants, or standard controls, were then allowed to bind to the precoated antibodies before washing and addition of a secondary HRP‐conjugated antibody. Absorbance was read at 450 nm by a plate reader and cytokine concentration was calculated from the standard curve.

## 3 RESULTS

### 3.1 Transcriptional regulation in MDM by LPS and IFNα

**Figure 2:**
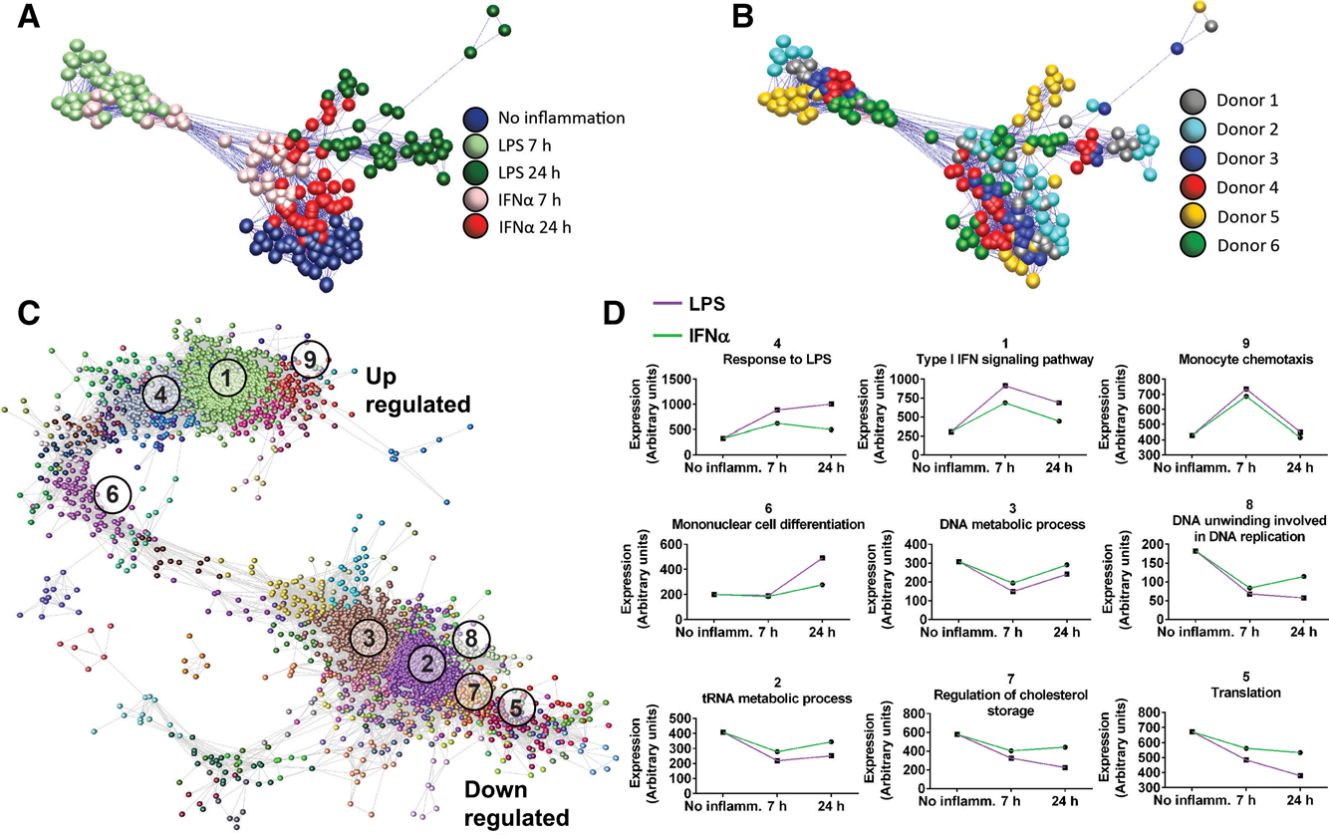
**Network analysis of the effects of inflammatory challenge and drug treatment on the transcriptome**. Sample‐sample correlation network constructed from the normalized microarray dataset colored according to (A) inflammatory stimulus or (B) by donor. Nodes represent samples within the dataset and edges represent correlations between samples greater than the threshold (*r* > 0.96). Samples closer to each other are more similar (highly correlated). Separation of the samples is observed by inflammatory stimulus and by donor. (C) A gene–gene correlation network constructed from DEGs from the microarray dataset (*r* > 0.93). Nodes represent genes which cluster together based on their pattern of expression. (D) Mean signal intensity of the genes in 9 of the largest clusters are displayed on the right with GO functional enrichment terms (numbered in order of size)

LPS significantly modulated the expression of 3294 genes and IFNα challenge 743 genes in human MDM, consistent with previous observations.^24^, ^27^, ^50^ Both challenges showed the greatest effect at 7 h (Fig. 1 and Supplemental Table S1). As previously observed in mouse macrophages,^28^ IFNα responsive genes were largely a subset of the LPS response: approximately 2 out of 3 of the IFNα responsive genes were also differentially expressed following LPS treatment. A sample‐to‐sample correlation network (Fig. 2) demonstrated that samples grouped together according to stimulus. Notably, the 7 h LPS samples were most distant (least correlated with) from the control samples. In keeping with evidence that LPS‐inducible genes in monocytes can be treated as quantitative traits with extensive variation between individuals,^51^ samples from the same donor tended to be in the same neighborhood.

A network model of the transcriptional network was constructed in which each of 3034 nodes represent a DEG and the edges connecting nodes represented a strong positive correlation (*r* > 0.93) between gene expression profiles across all the samples in the dataset (Fig. 2C). Genes with a similar expression profile tended to group closely together. Regulated genes grouped into 9 major clusters of strongly co‐expressed genes: 4 clusters of genes that were up‐regulated and 5 clusters of down‐regulated genes (Fig. 2D).

### 3.2 Effects of monoaminergic antidepressant drugs on whole transcriptome

At the nontoxic doses used in this study, neither antidepressant drug had a significant effect on the macrophage response to either LPS or IFNα (Supplemental Fig. S3).

### 3.3 Effects of anti‐inflammatory drugs on whole transcriptome

**Figure 3:**
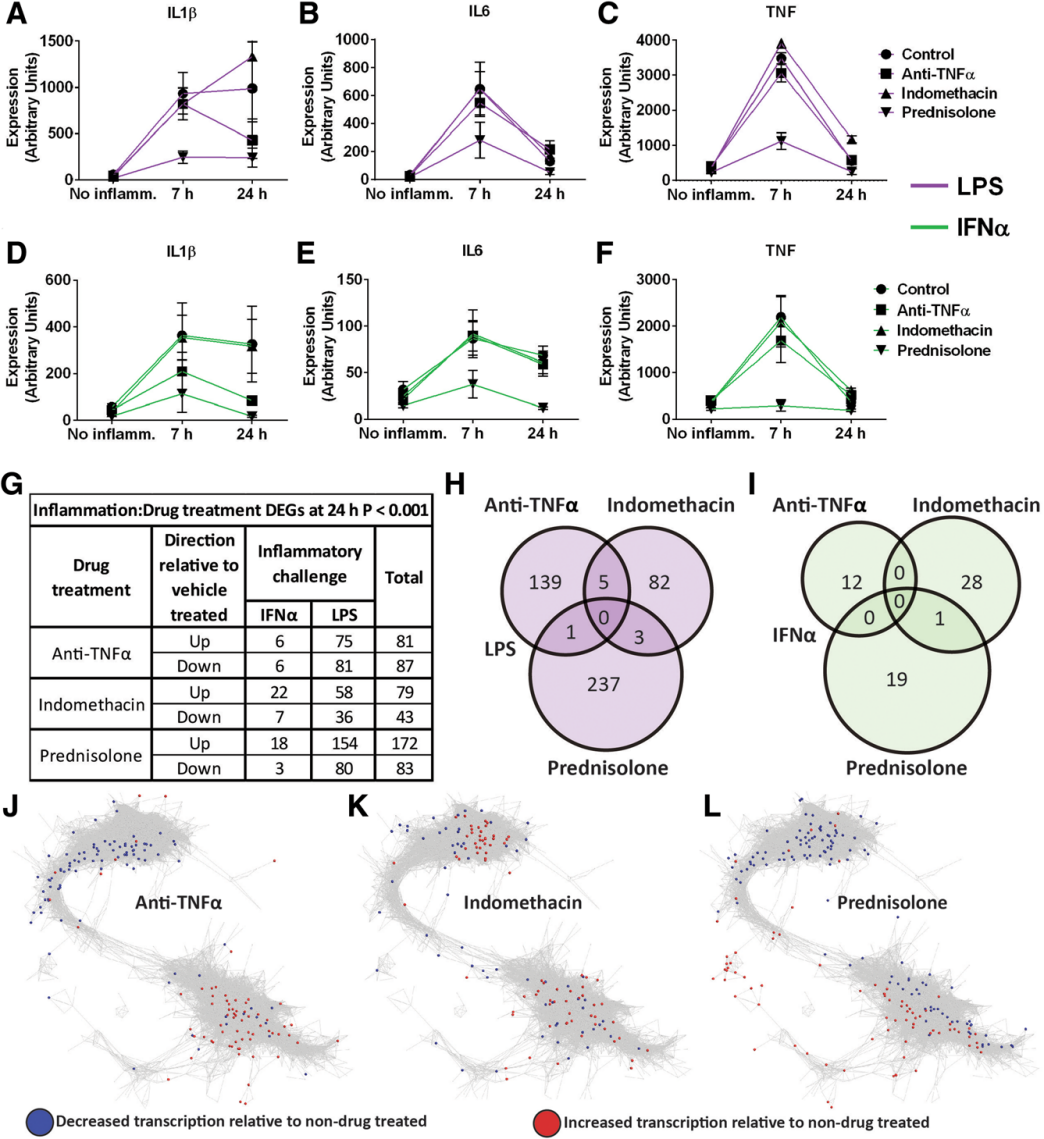
**Anti‐inflammatory drug effects on inflammation‐induced gene expression**. The expression intensity of *IL1β* (A, D), *IL6* (B, E), and *TNF* (C, F) are displayed following treatment with anti‐inflammatory drugs or vehicle control and LPS (A–C) or IFNα (D–F) challenge. The numbers of genes differentially expressed by the interaction of anti‐inflammatory drug treatment and proinflammatory challenge, and their direction of change when compared with control‐treated samples, are displayed in a table (G) and as Venn diagrams for each drug (H, I). These DEGs were overlaid on the gene–gene correlation network from Fig. 2: red = genes overexpressed by drug treatment, blue = genes underexpressed by drug treatment. Genes significantly modulated by anti‐TNFα treatment (J), indomethacin treatment (K), and prednisolone treatment (L) are displayed separately.

Consistent with their different modes of action, each of the anti‐inflammatory drugs influenced the response to LPS or IFNα in a distinct manner. For example, each drug had a specific effect on the transcription profile of 3 classical inflammatory genes (*IL1B, IL6*, and *TNF*) induced by LPS or IFNα challenge (Figs. 3A–F). These data were confirmed at the protein level as measurement of the corresponding cytokine in the supernatants of these same samples (Supplemental Fig. S4) reflected patterns observed at the gene level. A complete list of the DEGs associated with the responses to the two inflammatory stimuli, and the effect of each drug treatment is provided in Supplemental Table S2 and Fig. 3G, respectively. The Venn diagrams (Figs. 3H and I) further illustrate how each drug affected the inflammatory response in a unique manner. Drug modulated genes were visualized in the context of the gene network as a whole (Figs. 3J–L), demonstrating prednisolone and the anti‐TNF antibody act predominantly by reducing the expression of genes that were up‐regulated, or increasing the expression of genes that were down‐regulated by the proinflammatory stimuli. Enrichment analysis confirmed that the genes regulated by LPS or IFNα, and modulated by drug treatment, were functionally important for immune signaling and related to the mechanisms of drug action; see Supplemental Table S1 for a summary and Supplemental Table S3 for details. The effect of indomethacin was more complex than simply inhibiting the responses to LPS and IFNα, consistent with the known ability to prevent expression of prostaglandins, which act as feedback inhibitors of gene expression.^52^ In many cases, it appeared to further elevate the expression of genes already up‐ or down‐regulated, particularly those associated with lipid metabolism.

### 3.4 Regulated expression of genes involved in tryptophan metabolism

**Figure 4:**
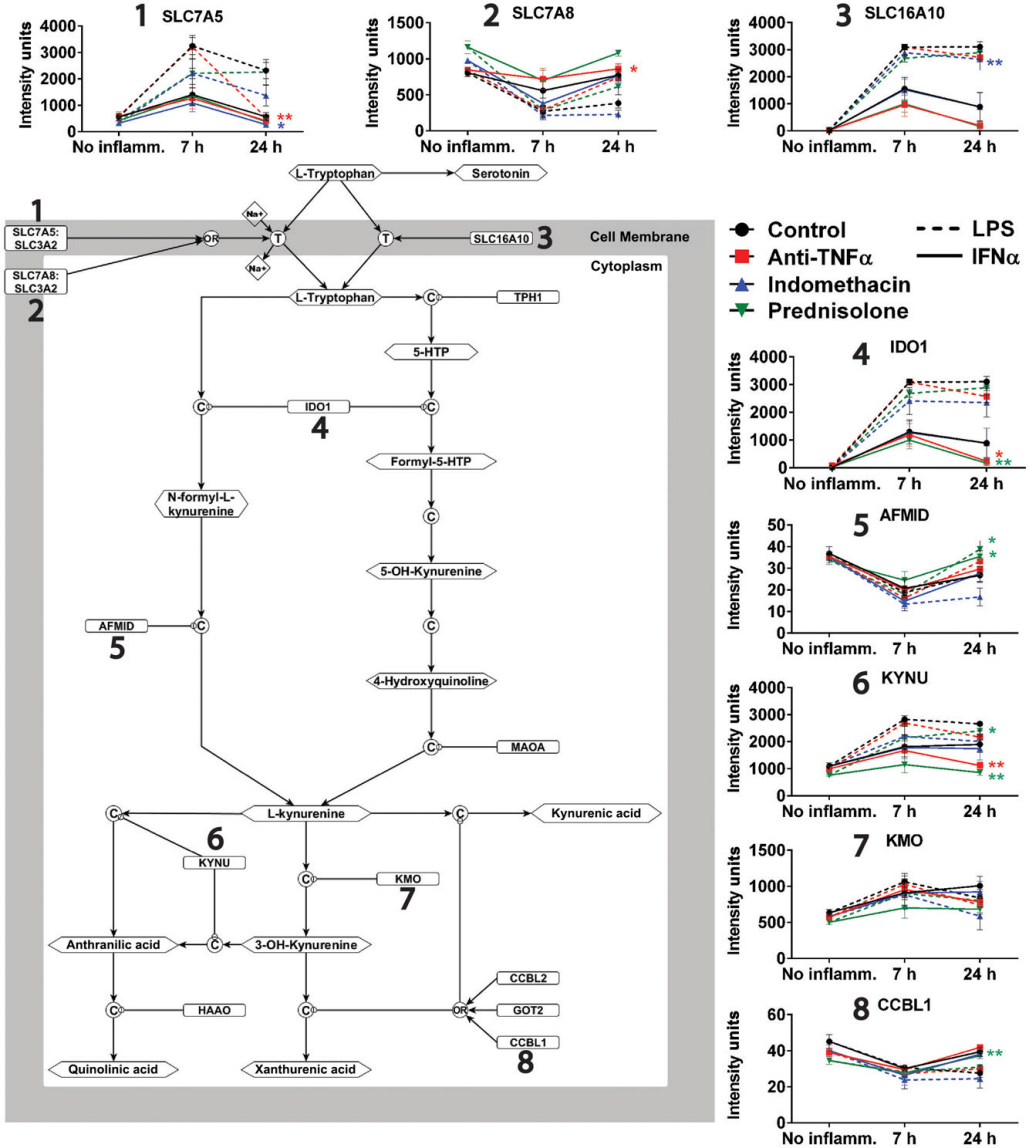
**Effects of proinflammatory challenge and anti‐inflammatory drugs on expression of genes related to tryptophan and kynurenine metabolism**. The pathway model summarizes the metabolic role of 8 proteins involved in tryptophan transport or catabolism as illustrated. The profile of expression changes for each gene following proinflammatory challenge is shown by the line graphs during each drug treatment. Significance is depicted for every significant interaction of anti‐inflammatory drug treatment with proinflammatory challenge, and color coded for each drug treatment. *P < 0.05, **P < 0.01

Amongst the many genes that demonstrated a significant interaction between the two inflammatory stimuli and drug treatment, we focused on pathways for tryptophan catabolism and transport. This pathway relates to the prior hypothesis that tryptophan metabolism could be a key mechanism linking peripheral inflammation to depression.^10^, ^20^, ^53^ A schematic representation of this metabolic pathway is illustrated in Fig. 4.

Both LPS and IFNα caused significant changes in the expression of genes encoding enzyme or transporter proteins that play crucial roles in the metabolism of tryptophan. LPS caused a pronounced up‐regulation of genes encoding 2 tryptophan transporters (*SLC16A10* and *SLC7A5*), and up‐regulation of genes coding enzymes on the pathway to kynurenine and its metabolites (*IDO1, KYNU*, and *KMO*). LPS also caused significant down‐regulation of genes coding kynurenine metabolic enzymes (*AFMID* and *CCBL7*). Other tryptophan transporters in human macrophages, the heavy chain Y+L SLC3A2 (CD98) and SLC7A7 are expressed constitutively at high levels.^27^ IFNα challenge resulted in a similar profile of effects on the expression of genes encoding the kynurenine metabolic enzymes, but did not produce significant elevation of the tryptophan transporter genes (Fig. 4).

Anti‐inflammatory drugs significantly modulated the on the inducible expression of genes encoding serotonin transporters and kynurenine metabolic enzymes. Interestingly, the direction of change in gene expression caused by anti‐inflammatory drug treatment was always opposite in sign to the change caused by activating stimulus. In other words, 4 of the 5 genes that were up‐regulated in response to challenge (*SLC16A10, SLC7A5, IDO1*, and *KYNU*), were less strongly induced after drug treatments; and all 3 genes that were down‐regulated in response to challenge (*SLC7A8, AFMID*, and *CCBL7*) were less strongly repressed (Fig. 4).

Thus, all anti‐inflammatory drugs tested were found to impact tryptophan‐ or kynurenine‐related gene expression. Indomethacin had effects mainly on tryptophan transporter genes; prednisolone on kynurenine metabolism genes; and anti‐TNFα on both class of genes.

### 3.5 Effects of macrophage activators (LPS, IFNα) and drugs on tryptophan‐related metabolite concentrations

**Figure 5:**
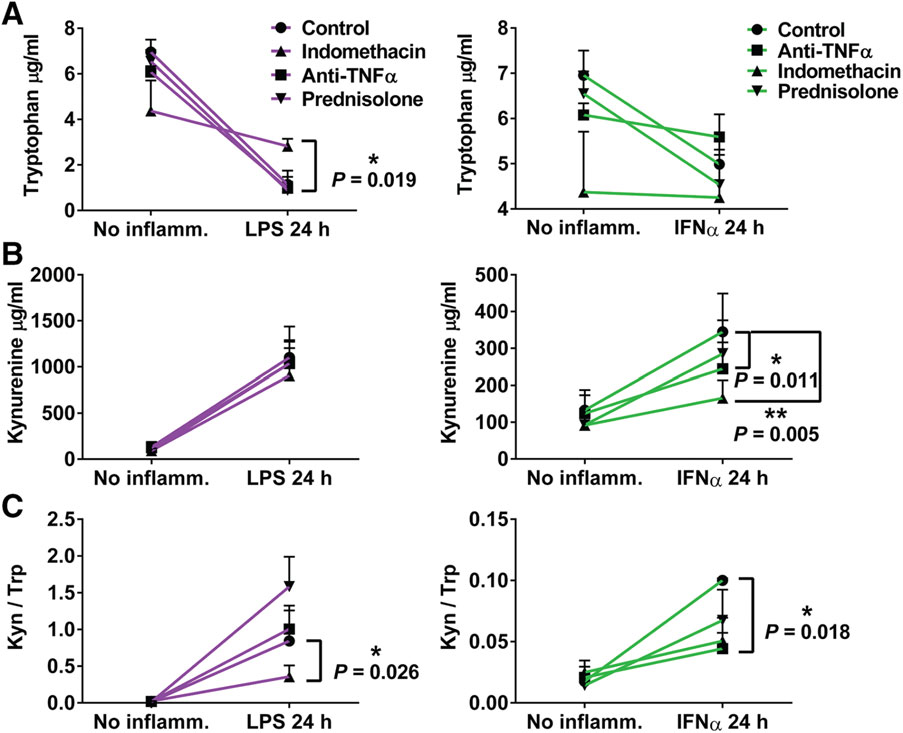
**Metabolomic analysis of effects of inflammatory challenge and anti‐inflammatory drug treatment on tryptophan concentration and tryptophan/kynurenine concentration ratio**. Tryptophan (A) and kynurenine (B) concentrations in cell culture supernatants 24 h after pretreatment of the 3 anti‐inflammatory drugs before each inflammatory challenge. (C) Ratio of kynurenine to tryptophan levels detected in the supernatant. Significance was calculated using a ratio paired *t* test. *P < 0.05 **P < 0.001

In keeping with the gene expression data, LPS treatment led to 70–80% depletion of tryptophan in the medium and accumulation of kynurenine. IFNα exerted a similar effect, but the magnitude of change in these metabolites was less. Of the three anti‐inflammatory drugs tested, only indomethacin significantly attenuated LPS‐induced reduction in tryptophan availability (Fig. 5). Both anti‐TNFα and indomethacin significantly reduced kynurenine production to ∼70% and ∼50% following IFNα stimulation but did not reduce kynurenine following LPS treatment (Fig. 5). The kynurenine to tryptophan (Kyn/Trp) ratio was used as a measure of tryptophan catabolism overall (Fig. 5). Anti‐TNFα treatment significantly moderated IFNα‐induced increases in Kyn/Trp (*P* = 0.018); and indomethacin treatment likewise significantly moderated LPS‐induced increases in the ratio of kynurenine to tryptophan (*P *= 0.026).

## 4 DISCUSSION

Evidence for a link between systemic inflammatory disease and depression (or depressive behavior in animals^3^, ^5^) is now overwhelming.^2^ We investigated the effects of LPS or IFNα challenge on gene expression by CSF1‐cultured MDMs. The scale of the transcriptional response to LPS and IFNα in these data was comparable to previous studies. LPS is itself known to induce type 1 IFNs (mainly IFNβ) in MDM, which act in an autocrine manner via IFNAR1, itself induced by LPS.^27^ Accordingly, the response to exogenous type 1 IFN (IFNα) was largely a subset of the LPS response, as reported in previous studies.^24^, ^28^, ^50^, ^51^, ^54^ None of the anti‐inflammatory agents we examined completely prevented the response to either challenge and, although prednisolone and anti‐TNFα both reduced expression of many genes that were up‐ or down‐regulated by the inflammatory challenges, each had a distinctive transcriptional effect. This diversity likely reflects the complex feed‐forward and negative feedback loops that characterize the response to LPS^27^, ^55^ and the different mode of action of each drug.

Both stimuli greatly increased expression (∼3000 fold following LPS) of the gene encoding the enzyme indoleamine 2,3‐dioxygenase (*IDO1*). IDO1 catalyzes the primary reaction in conversion of tryptophan to kynurenine; thereby reducing its availability as a precursor for serotonin metabolism. While this process has been associated with immunosuppression through Treg activation and effector T cell suppression,^56^ inflammation‐induced expression of this enzyme leads to tryptophan depletion both locally and systemically in chronic inflammatory states and has also been linked to alterations in mood.^5^, ^53^, ^57^ Similarly, the ability of host microbiota to control tryptophan metabolism has been functionally linked to influencing mood.^58^ Furthermore, polymorphisms in *IDO1* have been associated with susceptibility to IFNα‐induced depression in hepatitis patients.^59^ This may arise both from the depletion of tryptophan, and the generation of neurotoxic metabolites such as kynurenine.^5^, ^60^ However, tryptophan depletion in depressed patients was reportedly independent of kynurenine pathway activation.^61^

Anti‐TNFα reduced transcription of tryptophan catabolism enzymes, *IDO1* and *KYNU*, following IFNα stimulation but no significant effect of the drug was observed on these elements following LPS treatment. This is likely due to the transient induction of feed‐forward and negative feedback loops of TNFα transcription following inflammatory stimulus. Like IFNα, TNFα was predicted to be involved in an autocrine loop in MDM elicited by LPS, since the TNFα receptor is also induced.^27^ Our data (Supplemental Table S2) confirm and extend evidence of autocrine TNFα signaling as a feed‐forward activator of macrophage gene expression^30^, ^62^, ^63^ and identify the subset of inducible genes dependent upon that stimulus. The lack of impact of anti‐TNFα on LPS‐inducible *IDO1* may reflect the magnitude of the response. Alternatively, activation of IDO1 by IFN‐γ requires co‐stimulation by TNFα, which increases the occupancy of IFN‐response elements.^64^, ^65^ It may be that LPS provides this second signal independently of TNFα.^30^, ^62^, ^63^

A study examining the treatment of primary murine hippocampal cells with ibuprofen, a nonselective COX inhibitor like indomethacin, identified *TDO2* as the most significantly affected gene.^66^ TDO2, which is not expressed in macrophages, acts like IDO1 to metabolize tryptophan to kynurenic metabolites. However, although anti‐TNFα and prednisolone each significantly reduced *IDO1* activity at 24 h post‐IFNα stimulation, indomethacin treatment did not. Indomethacin did, however, significantly reduce transcription of tryptophan transporters induced by both inflammatory challenges, potentially reducing the availability of intracellular tryptophan as a substrate for IDO1. In contrast, anti‐TNFα treatment reduced transcription of one tryptophan transporter while increasing transcription of another during LPS challenge, and prednisolone did not affect the mRNA levels of any transporter.

To directly assess tryptophan uptake and catabolism following each treatment, we measured the concentrations of tryptophan and kynurenine in the supernatant. Indomethacin was the only inhibitor that impacted tryptophan levels during LPS challenge, likely due to a transcription repression of the tryptophan transporter. Prednisolone, while modulating expression of numerous inducible genes, did not produce any alteration in overall tryptophan catabolism (Supplemental Fig. S5), despite reducing expression of tryptophan catabolism‐related genes such *as IDO1, KYNU*, and *CCBL1*.

A recent review examined the anti‐inflammatory effects of antidepressant drugs.^67^ We found no evidence that either of the monoaminergic antidepressant drugs tested (TCA or SSRI) exerted any effect on the response in MDMs to LPS or IFNα. This is in contrast to reports that TCAs reduce proinflammatory signaling in phagocytes,^68^ and SSRIs have been reported to alter macrophage differentiation and inflammatory signaling.^67^, ^69^ SSRIs have also been reported to modulate glucocorticoid actions on monocytes.^70^ Although we found no direct effect of SSRIs or TCAs on macrophage responses to either LPS or IFNα, we did observe significant cytotoxicity at concentrations that were <20‐fold lower than circulating blood concentrations in treated patients. In support of this, nortriptyline has previously been shown to induce autophagy in macrophages.^39^ Indeed, many antidepressants are cationic amphipaths, and are therefore lysosomotropic^71^ likely accumulating selectively in phagocytic cells. Selective toxicity to macrophages may therefore contribute to the results reported in other studies, which used far higher concentrations of SSRIs than used here.^69^

There is already some evidence that anti‐inflammatory drugs can have antidepressant efficacy.^32^ Our results are compatible with the mechanistic interpretation that this may be at least partly attributable to the effects of anti‐inflammatory drugs on “normalization” of an inflammation‐induced bias in tryptophan and kynurenine metabolism. However, the results also indicate that this is unlikely to be the sole mechanism, and each agent may produce distinct patterns of regulation of genes that can impact indirectly on neuronal function expression. If each anti‐inflammatory agent is distinct in its actions, it may be that combinations would have novel potential efficacy.

## Supporting information

The antidepressants (top) act through blocking receptors for neuroactive agents. Escitalopram blocks the 5‐HT transporter preventing uptake and subsequent degradation of 5‐HT. Nortriptyline acts by blocking the histamine, cholinergic, muscarinic and β2‐adrenergic receptors and is believed to primarily block noradrenaline signaling, which results in less 5HT uptake. This results in a relative increase of 5‐HT available in the brain, thus improving mood. Inflammatory stimuli used in the assay, LPS and IFNα, are depicted at the bottom of the diagram. IFNα is recognised by its receptor inducing an inflammatory response and the production of proinflammatory cytokines such as TNFα. LPS is detected through the TLR4 complex and induces a powerful inflammatory response, which also involves production of TNFα and IFNβ which contribute to the overall response to LPS. Prednisolone (upper right) forms a complex with the glucocorticoid receptor and translocates to the nucleus. From here, the receptor ligand complex is known to up‐regulate anti‐inflammatory gene expression. In addition, it prevents inflammatory gene transcription via the NF‐κB transcription factor, RelA, in response to inflammatory stimuli such LPS or IFNα. TNFα is a powerful inflammatory cytokine, which is involved in autocrine signaling during inflammation, further driving the response. Anti‐TNFα antibodies (lower left) neutralise TNFα, preventing autocrine signaling and reducing further transcription of inflammatory mediators. The inflammatory response also up‐regulates prostaglandin synthases which further increase inflammation. Indomethacin inhibits prostaglandin synthases preventing inflammation from prostaglandin autocrine loops (right).

Fully differentiated hMDMs (day 8) were incubated for 24 h in various concentrations of the drugs indicated; nortriptyline **(A)**, escitalopram **(B)**, indomethacin **(C)** or prednisolone **(D)**. Cell viability was then assessed (**Supplementary Methods**).

**(A**) Number of differentially expressed genes (DEGs) between each condition versus control treated samples for each time point. **(B)** A correlation matrix constructed (*r* > 0.93) using only DEGs affected by either inflammatory stimulus or drug treatment (p < 0.001) where nodes represent genes. Cluster 8 was the only cluster which was significantly enriched for DEGs between control vs. antidepressant treated samples. **(C)** GO enrichment was performed on genes contained in cluster 8, together with the DEGs between antidepressant and control treated samples.

**Figure S4 Anti‐inflammatory drug effects on inflammation‐induced cytokine production**. The expression intensity of *IL6* (A, E) and *TNF* (B, F) are displayed along with cytokine production for IL‐6 (C, G) and TNF‐α (D, H) following treatment with anti‐inflammatory drugs or vehicle control and LPS (A – D) or *IFNα* (E – H) challenge.

The effects of anti‐inflammatory drug treatment on the tryptophan transport or catabolism components identified in Figure 3 are displayed for each gene as indicated. Signal intensity for each gene as calculated from the transcriptomic data is displayed on the y‐axis while the x‐axis represents values for the drug treatments and inflammatory challenges indicated for six donors. Significance was calculated using RM one‐way ANOVA, with the Greeenhouse‐Geisser correction, Holm‐Sidak's multiple comparison test, with individual variances computed for each comparison. *P < 0.05, **P < 0.01, ***P < 0.001.

Supporting Information

Supporting Information

Supporting Information

Supporting Information

## References

[1] Khandaker GM , Pearson RM , Zammit S , Lewis G , Jones PB . Association of serum interleukin 6 and C‐reactive protein in childhood with depression and psychosis in young adult life: A population‐based longitudinal study. JAMA Psychiatry. 2014;71:1121–1128. 25133871 10.1001/jamapsychiatry.2014.1332 PMC4561502

[2] Krishnadas R , Cavanagh J . Depression: An inflammatory illness?. J Neurol Neurosurg Psychiatry. 2012;83:495–502. 22423117 10.1136/jnnp-2011-301779

[3] Eisenberger NI , Berkman ET , Inagaki TK , Rameson LT , Mashal NM , Irwin MR . Inflammation‐induced anhedonia: Endotoxin reduces ventral striatum responses to reward. Biol Psychiatry. 2010;68:748–754. 20719303 10.1016/j.biopsych.2010.06.010 PMC3025604

[4] Remus JL , Dantzer R . Inflammation models of depression in rodents: Relevance to psychotropic drug discovery. Int J Neuropsychopharmacol. 2016;19:pyw028. 27026361 10.1093/ijnp/pyw028 PMC5043641

[5] Fuertig R , Azzinnari D , Bergamini G , et al. Mouse chronic social stress increases blood and brain kynurenine pathway activity and fear behaviour: Both effects are reversed by inhibition of indoleamine 2,3‐dioxygenase. Brain Behav Immun. 2016;54:59–72. 26724575 10.1016/j.bbi.2015.12.020

[6] Ignatova TM , Kinkul'kina MA , Morozov AO , Avdeeva TI , Volkov AV , Tikhonova Iu G . [Depressive disturbances during antiviral therapy in patients with type C hepatitis]. Klinicheskaia Med. 2007;85:58–63. 17882814

[7] Hepgul N , Cattaneo A , Agarwal K , et al. Transcriptomics in interferon‐alpha‐treated patients identifies inflammation‐, neuroplasticity‐ and oxidative stress‐related signatures as predictors and correlates of depression. Neuropsychopharmacology. 2016;41:2502–2511. 27067128 10.1038/npp.2016.50 PMC4983179

[8] Uguz F , Akman C , Kucuksarac S , Tufekci O . Anti‐tumor necrosis factor‐alpha therapy is associated with less frequent mood and anxiety disorders in patients with rheumatoid arthritis. Psychiatry Clin Neurosci. 2009;63:50–55. 19154212 10.1111/j.1440-1819.2008.01905.x

[9] Raison CL , Rutherford RE , Woolwine BJ , et al. A randomized controlled trial of the tumor necrosis factor antagonist infliximab for treatment‐resistant depression: The role of baseline inflammatory biomarkers. JAMA Psychiatry. 2013;70:31–41. 22945416 10.1001/2013.jamapsychiatry.4 PMC4015348

[10] Felger JC , Lotrich FE . Inflammatory cytokines in depression: Neurobiological mechanisms and therapeutic implications. Neuroscience. 2013;246:199–229. 23644052 10.1016/j.neuroscience.2013.04.060 PMC3741070

[11] Wichers MC , Koek GH , Robaeys G , Verkerk R , Scharpe S , Maes M . IDO and interferon‐alpha‐induced depressive symptoms: A shift in hypothesis from tryptophan depletion to neurotoxicity. Mol Psychiatry. 2005;10:538–544. 15494706 10.1038/sj.mp.4001600

[12] Ruhe HG , Mason NS , Schene AH . Mood is indirectly related to serotonin, norepinephrine and dopamine levels in humans: A meta‐analysis of monoamine depletion studies. Mol Psychiatry. 2007;12:331–359. 17389902 10.1038/sj.mp.4001949

[13] Capuron L , Ravaud A , Neveu PJ , Miller AH , Maes M , Dantzer R . Association between decreased serum tryptophan concentrations and depressive symptoms in cancer patients undergoing cytokine therapy. Mol Psychiatry. 2002;7:468–473. 12082564 10.1038/sj.mp.4000995

[14] Lee M , Ryu YH , Cho WG , et al. Relationship between dopamine deficit and the expression of depressive behavior resulted from alteration of serotonin system. Synapse. 2015;69:453–460. 26089169 10.1002/syn.21834

[15] Mechawar N , Savitz J . Neuropathology of mood disorders: Do we see the stigmata of inflammation?. Transl Psychiatry. 2016;6:e946. 27824355 10.1038/tp.2016.212 PMC5314124

[16] Cattaneo A , Ferrari C , Uher R , et al. Absolute measurements of macrophage migration inhibitory factor and interleukin‐1‐beta mRNA levels accurately predict treatment response in depressed patients. Int J Neuropsychopharmacol. 2016;19. 10.1093/ijnp/pyw045 PMC5091822 27207917

[17] Stahl SM . Mechanism of action of serotonin selective reuptake inhibitors. Serotonin receptors and pathways mediate therapeutic effects and side effects. J Affect Disord. 1998;51:215–235. 10333979 10.1016/s0165-0327(98)00221-3

[18] Szuster‐Ciesielska A , Tustanowska‐Stachura A , Slotwinska M , Marmurowska‐Michalowska H , Kandefer‐Szerszen M . In vitro immunoregulatory effects of antidepressants in healthy volunteers. Pol J Pharmacol. 2003;55:353–362. 14506314

[19] Dantzer R , O'Connor JC , Lawson MA , Kelley KW . Inflammation‐associated depression: From serotonin to kynurenine. Psychoneuroendocrinology. 2011;36:426–436. 21041030 10.1016/j.psyneuen.2010.09.012 PMC3053088

[20] Smith RS . The macrophage theory of depression. Med Hypoth. 1991;35:298–306. 10.1016/0306-9877(91)90272-z 1943879

[21] Hume DA , Summers KM , Rehli M . Transcriptional regulation and macrophage differentiation. Microbiol Spectrum. 2016;4. 10.1128/microbiolspec.MCHD-0024-2015 27337479

[22] Perry VH , Hume DA , Gordon S . Immunohistochemical localization of macrophages and microglia in the adult and developing mouse brain. Neuroscience. 1985;15:313–326. 3895031 10.1016/0306-4522(85)90215-5

[23] Brites D , Fernandes A . Neuroinflammation and depression: Microglia activation, extracellular microvesicles and microRNA dysregulation. Front Cell Neurosci. 2015;9:476. 26733805 10.3389/fncel.2015.00476 PMC4681811

[24] Kapetanovic R , Fairbairn L , Beraldi D , et al. Pig bone marrow‐derived macrophages resemble human macrophages in their response to bacterial lipopolysaccharide. J Immunol. 2012;188:3382–3394. 22393154 10.4049/jimmunol.1102649

[25] Takeuchi O , Akira S . Pattern recognition receptors and inflammation. Cell. 2010;140:805–820. 20303872 10.1016/j.cell.2010.01.022

[26] Satoh T , Akira S . Toll‐like receptor signaling and its inducible proteins. Microbiol Spectrum. 2016;4. 10.1128/microbiolspec.MCHD-0040-2016 28084212

[27] Baillie JK , Arner E , Daub C , et al. Analysis of the human monocyte‐derived macrophage transcriptome and response to lipopolysaccharide provides new insights into genetic aetiology of inflammatory bowel disease. PLoS Genet. 2017;13:e1006641. 28263993 10.1371/journal.pgen.1006641 PMC5358891

[28] Raza S , Barnett MW , Barnett‐Itzhaki Z , Amit I , Hume DA , Freeman TC . Analysis of the transcriptional networks underpinning the activation of murine macrophages by inflammatory mediators. J Leuk Biol. 2014;96:167–183. 10.1189/jlb.6HI0313-169R PMC4378362 24721704

[29] Emi Aikawa N , de Carvalho JF , Artur Almeida Silva C , Bonfa E . Immunogenicity of Anti‐TNF‐alpha agents in autoimmune diseases. Clin Rev Allergy Immunol. 2010;38:82–89. 19565360 10.1007/s12016-009-8140-3

[30] Huynh L , Kusnadi A , Park SH , Murata K , Park‐Min KH , Ivashkiv LB . Opposing regulation of the late phase TNF response by mTORC1‐IL‐10 signaling and hypoxia in human macrophages. Sci Rep. 2016;6:31959. 27558590 10.1038/srep31959 PMC4997257

[31] Ferreira SH , Moncada S , Vane JR . Indomethacin and aspirin abolish prostaglandin release from the spleen. Nat New Biol. 1971;231:237–239. 5284362 10.1038/newbio231237a0

[32] Miller AH , Raison CL . The role of inflammation in depression: From evolutionary imperative to modern treatment target. Nat Rev Immunol. 2016;16:22–34. 26711676 10.1038/nri.2015.5 PMC5542678

[33] Jubb A , Young R , Bickmore W , Hume D . Divergent transcriptional activation by glucocorticoids in mouse and human macrophages. Lancet. 2015;385(Suppl 1):S54. 10.1016/S0140-6736(15)60369-5 26312876

[34] Holgate ST , Polosa R . Treatment strategies for allergy and asthma. Nat Rev Immunol. 2008;8:218–230. 18274559 10.1038/nri2262

[35] Oh KS , Patel H , Gottschalk RA , et al. Anti‐inflammatory chromatinscape suggests alternative mechanisms of glucocorticoid receptor action. Immunity. 2017;47:298–309. e5. 28801231 10.1016/j.immuni.2017.07.012 PMC5572836

[36] Ahern GP . 5‐HT and the immune system. Curr Opin Pharmacol. 2011;11:29–33. 21393060 10.1016/j.coph.2011.02.004 PMC3144148

[37] O'Connell PJ , Wang X , Leon‐Ponte M , Griffiths C , Pingle SC , Ahern GP . A novel form of immune signaling revealed by transmission of the inflammatory mediator serotonin between dendritic cells and T cells. Blood. 2006;107:1010–1017. 16223770 10.1182/blood-2005-07-2903 PMC1895901

[38] Iskandar HN , Cassell B , Kanuri N , et al. Tricyclic antidepressants for management of residual symptoms in inflammatory bowel disease. J Clin Gastroenterol. 2014;48:423–429. 24406434 10.1097/MCG.0000000000000049 PMC4111227

[39] Sundaramurthy V , Barsacchi R , Samusik N , et al. Integration of chemical and RNAi multiparametric profiles identifies triggers of intracellular mycobacterial killing. Cell Host Microbe. 2013;13:129–142. 23414754 10.1016/j.chom.2013.01.008

[40] Francois C , Descamps V , Brochot E , et al. Relationship between the hepatitis C viral load and the serum interferon concentration during the first week of peginterferon‐alpha‐2b‐ribavirin combination therapy. J Med Virol. 2010;82:1640–1646. 20827759 10.1002/jmv.21837

[41] Carlsson B , Holmgren A , Ahlner J , Bengtsson F . Enantioselective analysis of citalopram and escitalopram in postmortem blood together with genotyping for CYP2D6 and CYP2C19. J Anal Toxicol. 2009;33:65–76. 19239731 10.1093/jat/33.2.65

[42] Martinez MA , Sanchez de la Torre C , Almarza E . A comparative solid‐phase extraction study for the simultaneous determination of fluoxetine, amitriptyline, nortriptyline, trimipramine, maprotiline, clomipramine, and trazodone in whole blood by capillary gas‐liquid chromatography with nitrogen‐phosphorus detection. J Anal Toxicol. 2003;27:353–358. 14516488 10.1093/jat/27.6.353

[43] Gero D , Szoleczky P , Modis K , et al. Identification of pharmacological modulators of HMGB1‐induced inflammatory response by cell‐based screening. PloS One. 2013;8:e65994. 23799067 10.1371/journal.pone.0065994 PMC3682954

[44] Xu K , Yen T , Geczy CL . Il‐10 up‐regulates macrophage expression of the S100 protein S100A8. J Immunol. 2001;166:6358–6366. 11342660 10.4049/jimmunol.166.10.6358

[45] Kauffmann A , Gentleman R , Huber W . arrayQualityMetrics–a bioconductor package for quality assessment of microarray data. Bioinformatics. 2009;25:415–416. 19106121 10.1093/bioinformatics/btn647 PMC2639074

[46] Gautier L , Cope L , Bolstad BM , Irizarry RA . affy–analysis of Affymetrix GeneChip data at the probe level. Bioinformatics. 2004;20:307–315. 14960456 10.1093/bioinformatics/btg405

[47] Ritchie ME , Phipson B , Wu D , et al. limma powers differential expression analyses for RNA‐sequencing and microarray studies. Nucleic Acids Res. 2015;43:e47. 25605792 10.1093/nar/gkv007 PMC4402510

[48] Irizarry RA , Hobbs B , Collin F , et al. Exploration, normalization, and summaries of high density oligonucleotide array probe level data. Biostatistics. 2003;4:249–264. 12925520 10.1093/biostatistics/4.2.249

[49] Fabregat A , Sidiropoulos K , Garapati P , et al. The reactome pathway knowledgebase. Nucleic Acids Res. 2016;44:D481–7. 26656494 10.1093/nar/gkv1351 PMC4702931

[50] Decker T , Muller M , Stockinger S . The yin and yang of type I interferon activity in bacterial infection. Nat Rev Immunol. 2005;5:675–687. 16110316 10.1038/nri1684

[51] Fairfax BP , Humburg P , Makino S , et al. Innate immune activity conditions the effect of regulatory variants upon monocyte gene expression. Science. 2014;343:1246949. 24604202 10.1126/science.1246949 PMC4064786

[52] Desanctis JB , Varesio L , Radzioch D . Prostaglandins inhibit lipoprotein lipase gene expression in macrophages. Immunology. 1994;81:605–610. 8039811 PMC1422386

[53] Fuchs D , Moller AA , Reibnegger G , Stockle E , Werner ER , Wachter H . Decreased serum tryptophan in patients with HIV‐1 infection correlates with increased serum neopterin and with neurologic/psychiatric symptoms. J Acquir Immune Defic Syndr. 1990;3:873–876. 2166783

[54] Schroder K , Irvine KM , Taylor MS , et al. Conservation and divergence in Toll‐like receptor 4‐regulated gene expression in primary human versus mouse macrophages. Proc Natl Acad Sci U S A. 2012;109:E944–53. 22451944 10.1073/pnas.1110156109 PMC3341041

[55] Wells CA , Ravasi T , Hume DA . Inflammation suppressor genes: Please switch out all the lights. J Leuk Biol. 2005;78:9–13. 10.1189/jlb.1204710 15774547

[56] Soliman H , Mediavilla‐Varela M , Antonia S . Indoleamine 2,3‐dioxygenase: Is it an immune suppressor?. Cancer J. 2010;16:354–359. 20693847 10.1097/PPO.0b013e3181eb3343 PMC3850167

[57] Cozzi A , Zignego AL , Carpendo R , et al. Low serum tryptophan levels, reduced macrophage IDO activity and high frequency of psychopathology in HCV patients. J Viral Hepat. 2006;13:402–408. 16842443 10.1111/j.1365-2893.2005.00706.x

[58] O'Mahony SM , Clarke G , Borre YE , Dinan TG , Cryan JF . Serotonin, tryptophan metabolism and the brain‐gut‐microbiome axis. Behav Brain Res. 2015;277:32–48. 25078296 10.1016/j.bbr.2014.07.027

[59] Smith AK , Simon JS , Gustafson EL , et al. Association of a polymorphism in the indoleamine‐ 2,3‐dioxygenase gene and interferon‐alpha‐induced depression in patients with chronic hepatitis C. Mol Psychiatry. 2012;17:781–789. 21691274 10.1038/mp.2011.67 PMC3179823

[60] Zhou W , Dantzer R , Budac DP , et al. Peripheral indoleamine 2,3‐dioxygenase 1 is required for comorbid depression‐like behavior but does not contribute to neuropathic pain in mice. Brain Behav Immun. 2015;46:147–153. 25637485 10.1016/j.bbi.2015.01.013 PMC4414738

[61] Hughes MM , Carballedo A , McLoughlin DM , et al. Tryptophan depletion in depressed patients occurs independent of kynurenine pathway activation. Brain Behav Immun. 2012;26:979–987. 22683764 10.1016/j.bbi.2012.05.010

[62] Yarilina A , Park‐Min KH , Antoniv T , Hu X , Ivashkiv LB . TNF activates an IRF1‐dependent autocrine loop leading to sustained expression of chemokines and STAT1‐dependent type I interferon‐response genes. Nat Immunol. 2008;9:378–387. 18345002 10.1038/ni1576

[63] Caldwell AB , Cheng Z , Vargas JD , Birnbaum HA , Hoffmann A . Network dynamics determine the autocrine and paracrine signaling functions of TNF. Genes Dev. 2014;28:2120–2133. 25274725 10.1101/gad.244749.114 PMC4180974

[64] Robinson CM , Shirey KA , Carlin JM . Synergistic transcriptional activation of indoleamine dioxygenase by IFN‐gamma and tumor necrosis factor‐alpha. J Interf Cytokine Res. 2003;23:413–421. 10.1089/107999003322277829 PMC1488822 13678429

[65] Konan KV , Taylor MW . Importance of the two interferon‐stimulated response element (ISRE) sequences in the regulation of the human indoleamine 2,3‐dioxygenase gene. J Biol Chem. 1996;271:19140–19145. 8702590 10.1074/jbc.271.32.19140

[66] Woodling NS , Colas D , Wang Q , et al. Cyclooxygenase inhibition targets neurons to prevent early behavioural decline in Alzheimer's disease model mice. Brain. 2016;139:2063–2081. 10.1093/brain/aww117 PMC4939702 27190010

[67] Kalkman HO , Feuerbach D . Antidepressant therapies inhibit inflammation and microglial M1‐polarization. Pharmacol Therap. 2016;163:82–93. 27101921 10.1016/j.pharmthera.2016.04.001

[68] Gosain A , Gamelli RL , DiPietro LA . Norepinephrine‐mediated suppression of phagocytosis by wound neutrophils. J Surg Res. 2009;152:311–318. 18952237 10.1016/j.jss.2008.05.001 PMC2683017

[69] Durairaj H , Steury MD , Parameswaran N . Paroxetine differentially modulates LPS‐induced TNFalpha and IL‐6 production in mouse macrophages. Int Immunopharmacol. 2015;25:485–492. 25744603 10.1016/j.intimp.2015.02.029 PMC4373999

[70] Carvalho LA , Garner BA , Dew T , Fazakerley H , Pariante CM . Antidepressants, but not antipsychotics, modulate GR function in human whole blood: An insight into molecular mechanisms. Eur Neuropsychopharmacol. 2010;20:379–387. 20231081 10.1016/j.euroneuro.2010.02.006 PMC2982744

[71] Chen J , Korostyshevsky D , Lee S , Perlstein EO . Accumulation of an antidepressant in vesiculogenic membranes of yeast cells triggers autophagy. PloS One. 2012;7:e34024. 22529904 10.1371/journal.pone.0034024 PMC3329523

