# Supplementary figures and images for: Effects of anti‐inflammatory drugs on the expression of tryptophan‐metabolism genes by human macrophages
Source: J Leukoc Biol. 2018 Jan 26;103(4):681–92. doi: 10.1002/JLB.3A0617-261R (PMC5918594; doi:10.1002/JLB.3A0617-261R)

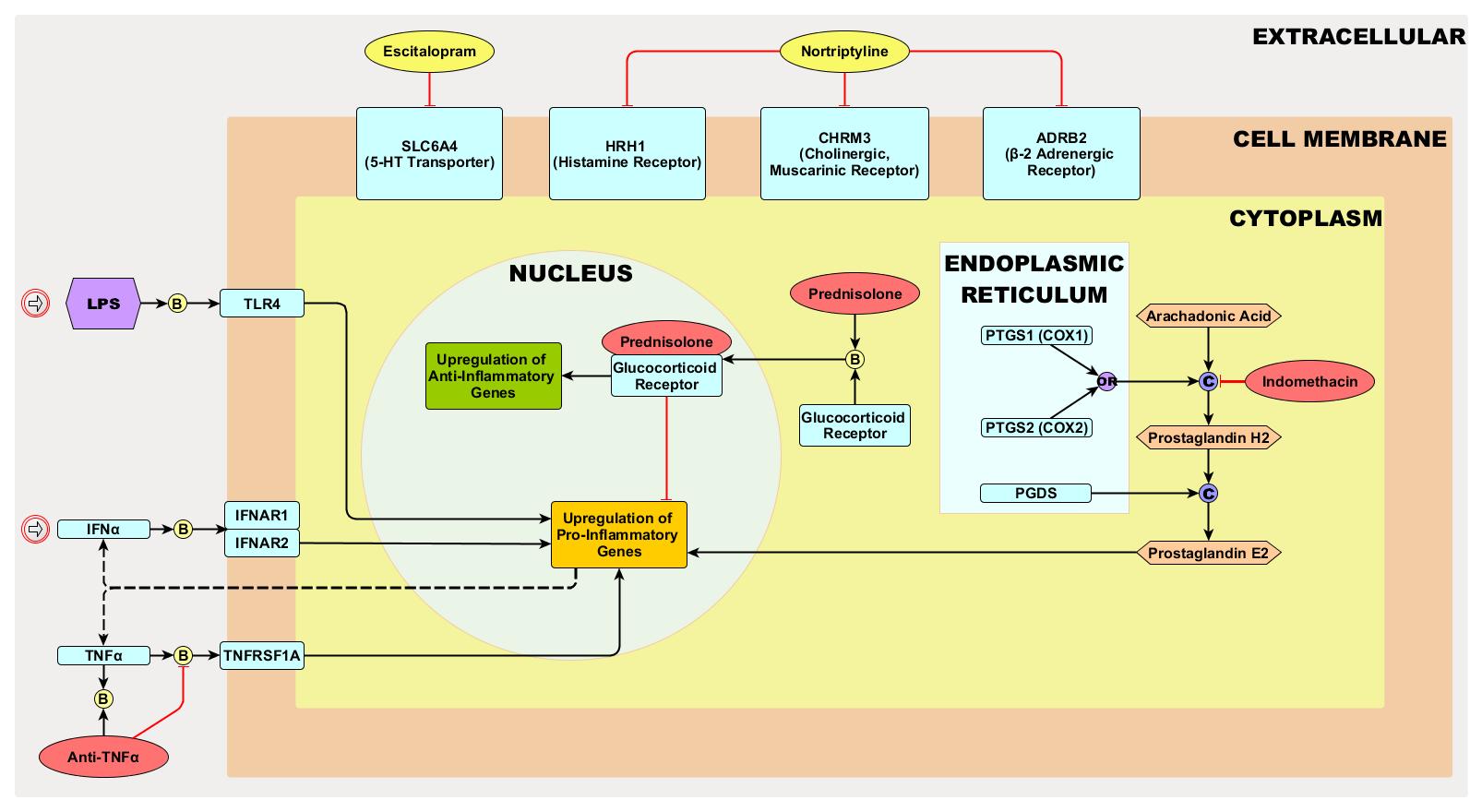

Supplement: Supplementary file 1 — The antidepressants (top) act through blocking receptors for neuroactive agents. Escitalopram blocks the 5‐HT transporter preventing uptake and subsequent degradation of 5‐HT. Nortriptyline acts by blocking the histamine, cholinergic, muscarinic and β2‐adrenergic receptors and is believed to primarily block noradrenaline signaling, which results in less 5HT uptake. This results in a relative increase of 5‐HT available in the brain, thus improving mood. Inflammatory stimuli used in the assay, LPS and IFNα, are depicted at the bottom of the diagram. IFNα is recognised by its receptor inducing an inflammatory response and the production of proinflammatory cytokines such as TNFα. LPS is detected through the TLR4 complex and induces a powerful inflammatory response, which also involves production of TNFα and IFNβ which contribute to the overall response to LPS. Prednisolone (upper right) forms a complex with the glucocorticoid receptor and translocates to the nucleus. From here, the receptor ligand complex is known to up‐regulate anti‐inflammatory gene expression. In addition, it prevents inflammatory gene transcription via the NF‐κB transcription factor, RelA, in response to inflammatory stimuli such LPS or IFNα. TNFα is a powerful inflammatory cytokine, which is involved in autocrine signaling during inflammation, further driving the response. Anti‐TNFα antibodies (lower left) neutralise TNFα, preventing autocrine signaling and reducing further transcription of inflammatory mediators. The inflammatory response also up‐regulates prostaglandin synthases which further increase inflammation. Indomethacin inhibits prostaglandin synthases preventing inflammation from prostaglandin autocrine loops (right). [file JLB-103-681-s001.jpg]

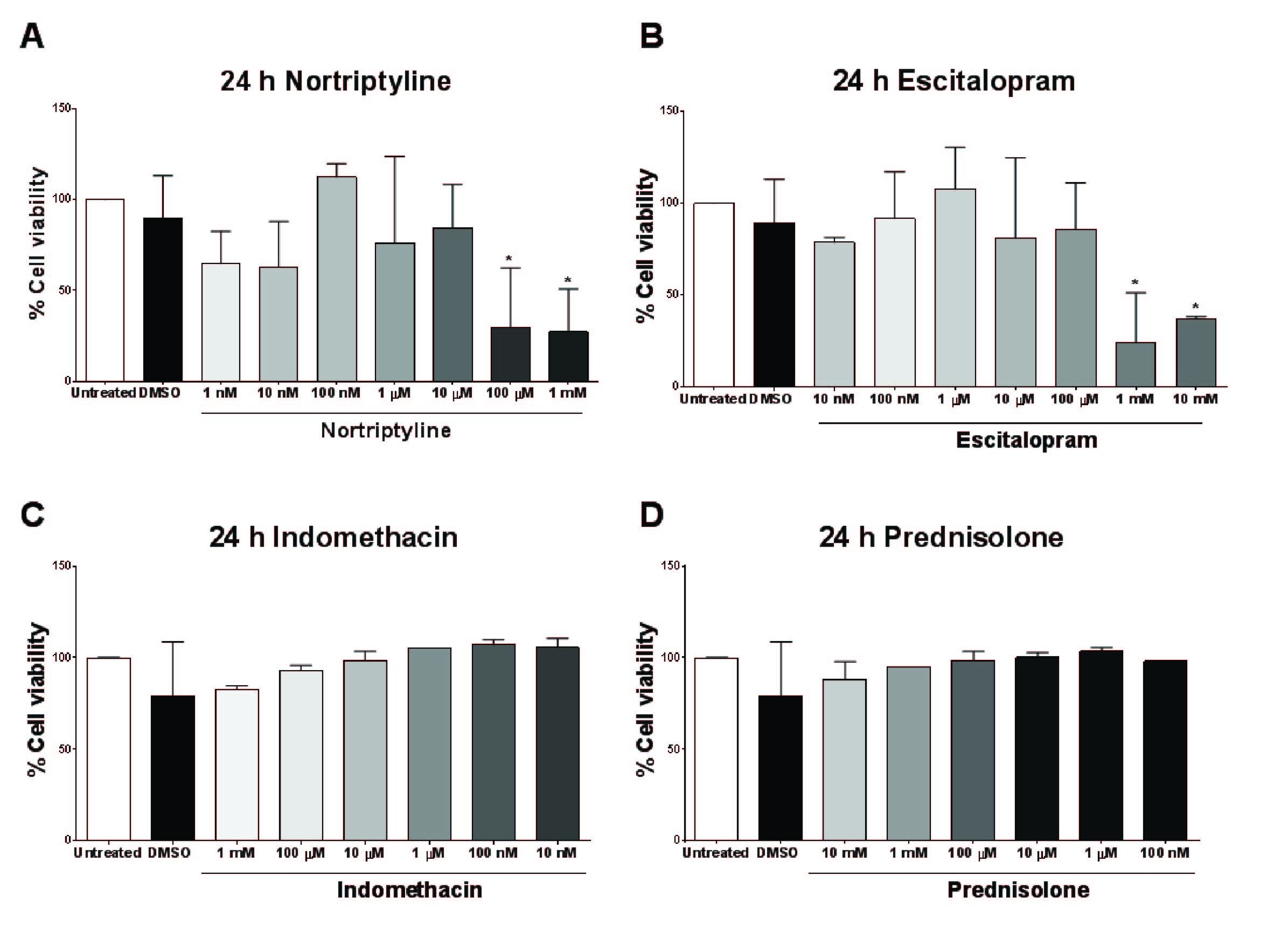

Supplement: Supplementary file 2 — Fully differentiated hMDMs (day 8) were incubated for 24 h in various concentrations of the drugs indicated; nortriptyline (A), escitalopram (B), indomethacin (C) or prednisolone (D). Cell viability was then assessed (Supplementary Methods). [file JLB-103-681-s002.jpg]

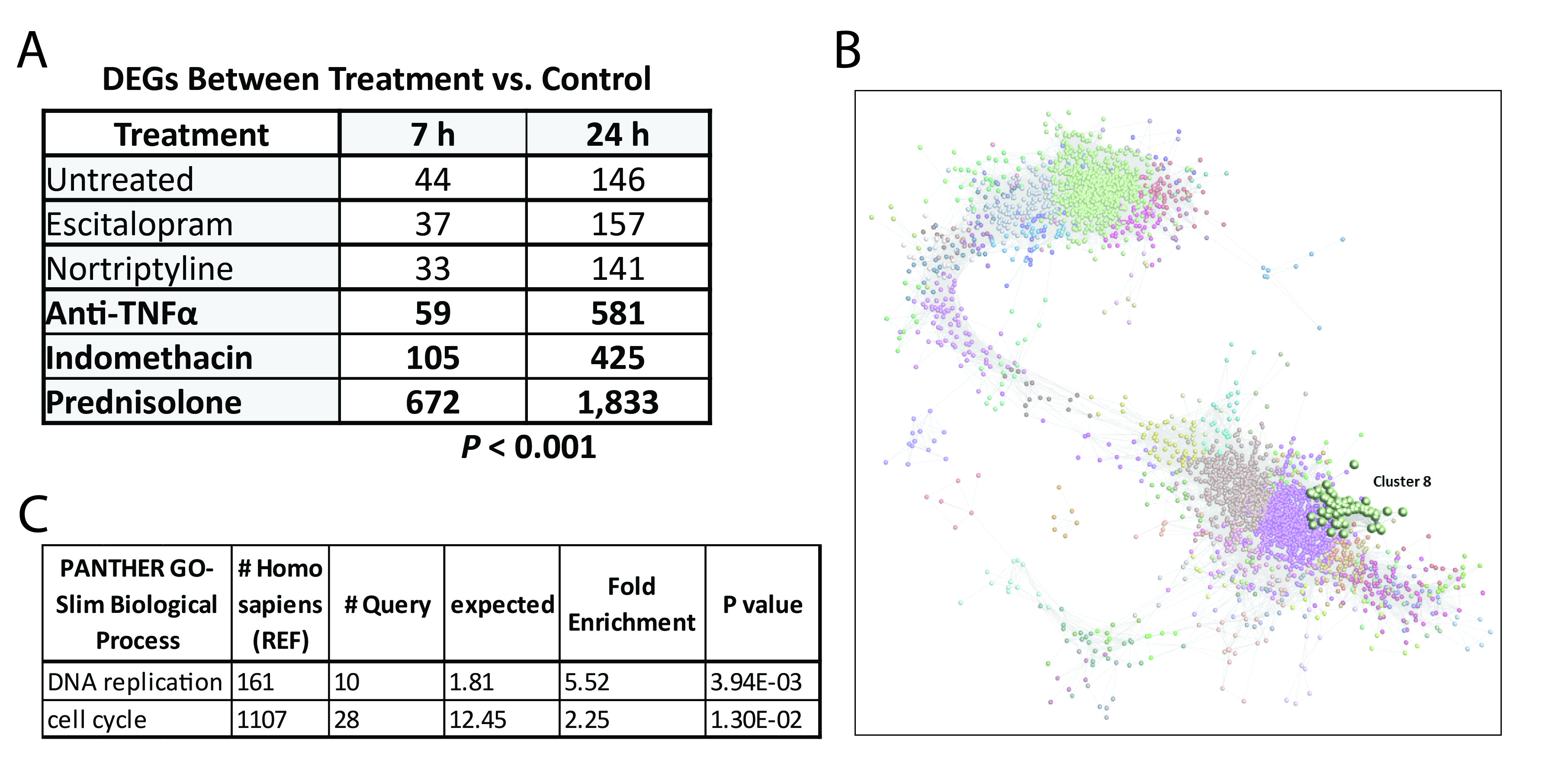

Supplement: Supplementary file 3 — (A) Number of differentially expressed genes (DEGs) between each condition versus control treated samples for each time point. (B) A correlation matrix constructed (r > 0.93) using only DEGs affected by either inflammatory stimulus or drug treatment (p < 0.001) where nodes represent genes. Cluster 8 was the only cluster which was significantly enriched for DEGs between control vs. antidepressant treated samples. (C) GO enrichment was performed on genes contained in cluster 8, together with the DEGs between antidepressant and control treated samples. [file JLB-103-681-s003.jpg]

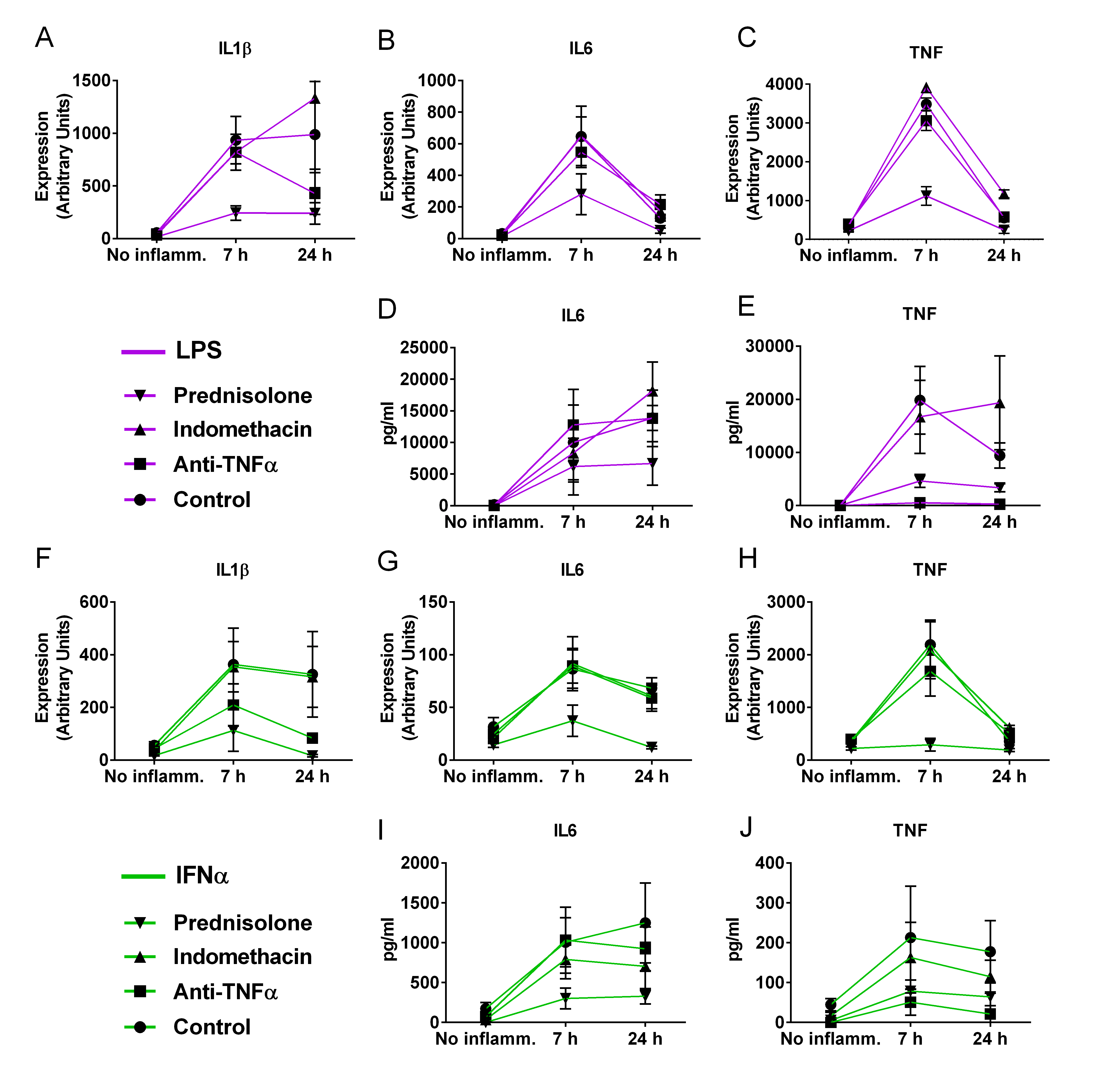

Supplement: Supplementary file 4 — Figure S4 Anti‐inflammatory drug effects on inflammation‐induced cytokine production. The expression intensity of IL6 (A, E) and TNF (B, F) are displayed along with cytokine production for IL‐6 (C, G) and TNF‐α (D, H) following treatment with anti‐inflammatory drugs or vehicle control and LPS (A – D) or IFNα (E – H) challenge. [file JLB-103-681-s004.jpg]

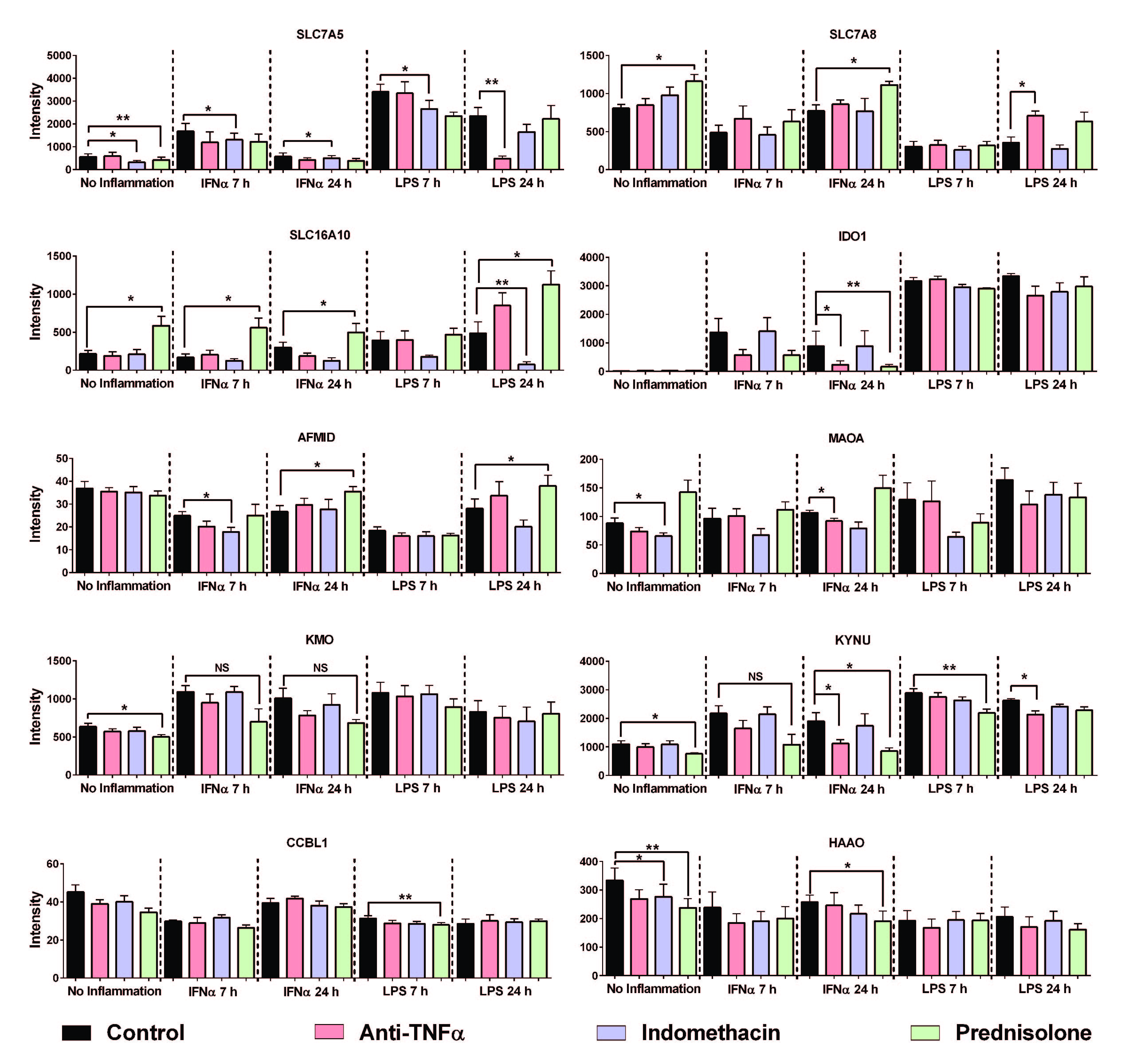

Supplement: Supplementary file 5 — The effects of anti‐inflammatory drug treatment on the tryptophan transport or catabolism components identified in Figure 3 are displayed for each gene as indicated. Signal intensity for each gene as calculated from the transcriptomic data is displayed on the y‐axis while the x‐axis represents values for the drug treatments and inflammatory challenges indicated for six donors. Significance was calculated using RM one‐way ANOVA, with the Greeenhouse‐Geisser correction, Holm‐Sidak's multiple comparison test, with individual variances computed for each comparison. *P < 0.05, **P < 0.01, ***P < 0.001. [file JLB-103-681-s005.jpg]
