# Supplementary material for: Effects of anti‐inflammatory drugs on the expression of tryptophan‐metabolism genes by human macrophages
Source: J Leukoc Biol. 2018 Jan 26;103(4):681–92. doi: 10.1002/JLB.3A0617-261R (PMC5918594; doi:10.1002/JLB.3A0617-261R)
Supplement: Supplementary file 9 — Supporting Information [file JLB-103-681-s009.docx]

**Supplementary Materials and Methods**

**Optimisation of inflammatory stimulation and drug concentrations**

Cell viability was in part used to optimise the concentrations of drugs used (other than anti-TNFα). This was performed using CellTiter-Glo (Promega Ltd, Southampton, UK) according to manufacturer’s instructions. Briefly, cells were seeded at 1x10^5^ cells/well in 96-well, flat-bottomed white-walled plates (Corning Ltd., Wiesbaden, Germany) and cultured in 100 µl media. Following maturation and consequent drug treatment, 100 µl CellTiter-Glo was added to each well (1:1 ratio) and the plate was placed on a shaker for 10 min. Luminescence was measured in technical triplicates using a GloMax®96- Microplate Luminometer (Promega) and percentage viability of each treatment was made relative to the untreated control. For escitalopram and nortriptyline, six different donors were used, three for indomethacin and prednisolone treatments. Fig. S2 A and B display the cell viability in the presence of escitalopram (SSRI) or nortriptyline (TCA) at 24 h post-treatment. To ensure maximal drug contact with the cells without affecting viability, concentrations of 100 nM for nortriptyline and 1 µM for escitalopram were selected for the transcriptomics assay. These concentrations are higher than peak whole blood concentrations of escitalopram (~0.1 nM (Carlsson *et al*, 2009)) or nortriptyline (50 nM (Martinez *et al*, 2003)) in patients. Fig. S2C and D shows cell viability was found not to be significantly affected by any concentrations of indomethacin or prednisolone tested. We therefore selected concentrations of 100 µM indomethacin and 1 µM prednisolone, as used in previous *in vitro* studies of macrophages (Gero *et al*, 2013; Xu *et al*, 2001). The amount of anti-TNFα antibody used was calculated to be 5 µg/ml. This was based on the suppliers ND_50_ value of 0.01-0.04 µg/ml in the presence of 0.75 ng/ml TNFα and our own measurements of maximum TNFα production from stimulated macrophages (<30 ng/ml). We then used to the following equation to calculate the amount of antibody required: $K_{D} = \frac{\left[ mAb \right][ Target]}{[Complex]}$. Fig. S1 illustrates the known mode of action of each drug on peripheral macrophage cells.

The final parameters for the experiment involving MDM, inflammatory stimuli and drug treatments are outlined in Fig. 1A. Each of the MDM cultures from six different individuals were treated separately with each of the three anti-inflammatory drugs, two antidepressants, vehicle control or left untreated. Samples were then stimulated with either IFNα or LPS for either 7 or 24 h, or incubated for 24 h with no inflammatory stimulus as a control. RNA was extracted from the samples and analysed by expression microarray

**Data Analysis of Expression data using Miru**

A table of the normalised microarray data, containing only intensity values for each DEG (p < 0.001) across the dataset over any of the drug or inflammatory treatments, was loaded into Miru (Kajeka Ltd., Edinburgh). Following annotation of each sample within the dataset, the correlation between samples was examined initially in a sample/sample correlation plot. For the gene/gene correlation studies, a range of correlation coefficients and MCL values were then used to determine an optimal graph structure containing as many nodes with minimal edges, from which gene clusters were defined. The statistical analysis results were used to annotate genes and mapped onto the networks. This allowed us to view the distribution and interactions of each statistically significant gene between other significant genes, including adjacent non-significant genes with which they shared a high level of correlation across the dataset. This gave a more enriched and holistic view of the transcriptional outcomes of the induced effect (drug/inflammatory stimulation).

**Quantitation of selected neurotransmitter metabolites**

Supernatants of each of the samples analysed by microarray were used for metabolic analyses. 2 μl of a 10 ng/μl solution of caffeic acid was added to 125 μl of supernatant, which acted as an internal standard to control for variations in extraction, and 500 μl 4°C acetonitrile. Samples were vortexed and the precipitate removed by centrifugation (13,000 xg for 15 min at 4°C). The supernatant was evaporated under vacuum and the residue resuspended in 25 μl of labelling buffer (100 mM sodium tetraborate). 25 μl of labelling reagent (2% (v/v) benzoyl chloride in acetonitrile) was added and the reaction mixed at room temperature. Insoluble material was removed by centrifugation (13,000 x g for 10 min at 4°C) and the supernatant analysed by HPLC-MS (All reagents from Sigma-Aldrich).

Neurochemicals and tryptophan metabolites were separated by use of an Ascentis Express HPLC column (C18, id 2.1 mm x length 15 cm, 2.7 μm bead size, Supelco, Sigma-Aldrich) attached to a Dionex Ultimate HPLC system. The eluent was pumped through the column at a flow rate of 200 μl/min (solvent A was 10 mM ammonium formate and 0.15% formic acid, solvent B was acetonitrile) and the separation was developed by a multipart gradient as follows: 0 min – 20% B; 0.5 min – 20% B; 2 min – 45% B; 7.5 min – 64% B; 8 min – 100% B. This gradient was followed by a 3.5 minute hold at 100% B, followed by a reduction to 20% B and a 1 min hold to re-equilibrate the column. The eluent was passed directly to the electrospray source of an Amazon ETD ion trap mass spectrometer (Bruker, Coventry, UK) operated in +ve ion, multiple reaction monitoring mode. At least 6-point standard curves of pre-labelled analytes in solvent A were built and samples were run in at least duplicate, in four batches. Data processing was performed using the Bruker Compass plugin, QuantAnalysis. Any effect of matrix on measurement of neurochemicals in the brain samples was corrected for by use of measured concentration of the internal standard, caffeic acid. Finally, repeat measurements were averaged, and significant differences in the concentration of different analytes were revealed by student t-tests.
